# Thermal degradation of Affinisol HPMC: Optimum Processing Temperatures for Hot Melt Extrusion and 3D Printing

**DOI:** 10.1007/s11095-023-03592-z

**Published:** 2023-08-23

**Authors:** Roman Svoboda, Marie Nevyhoštěná, Jana Macháčková, Jan Vaculík, Kateřina Knotková, Maria Chromčíková, Alena Komersová

**Affiliations:** 1 Department of Physical Chemistry, Faculty of Chemical Technology, University of Pardubice, Studentská 573, 532 10 Pardubice, Czech Republic; 2 VILA – Joined Glass Centre of the IIC SAS, TnUAD, FChPT STU, Študentská 2, SK-911 50 Trenčín, Slovakia; 3 FunGlass, Alexander Dubček University of Trenčín, Študentská 2, SK-911 50 Trenčín, Slovakia

**Keywords:** affinisol, DSC, hot melt extrusion, TGA, thermal degradation

## Abstract

**Purpose:**

Affinisol HPMC HME is a new popular form of hypromellose specifically designed for the hot melt extrusion and 3D printing of pharmaceutical products. However, reports of its thermal stability include only data obtained under inert N_2_ atmosphere, which is not consistent with the common pharmaceutical practice. Therefore, detailed investigation of its real-life thermal stability in air is paramount for identification of potential risks and limitations during its high-temperature processing.

**Methods:**

In this work, the Affinisol HPMC HME 15LV powder as well as extruded filaments will be investigated by means of thermogravimetry, differential scanning calorimetry and infrared spectroscopy with respect to its thermal stability.

**Results:**

The decomposition in N_2_ was proceeded in accordance with the literature data and manufacturer’s specifications: onset at ~260°C at 0.5°C·min^−1^, single-step mass loss of 90–95%. However, in laboratory or industrial practice, high-temperature processing is performed in the air, where oxidation-induced degradation drastically changes. The thermogravimetric mass loss in air proceeded in three stages: ~ 5% mass loss with onset at 150°C, ~ 70% mass loss at 200°C, and ~ 15% mass loss at 380°C. Diffusion of O_2_ into the Affinisol material was identified as the rate-determining step.

**Conclusion:**

For extrusion temperatures ≥170°C, Affinisol exhibits a significant degree of degradation within the 5 min extruder retention time. Hot melt extrusion of pure Affinisol can be comfortably performed below this temperature. Utilization of plasticizers may be necessary for safe 3D printing.

## Introduction

A massive increase in new discoveries and syntheses of small molecule drugs has been achieved in recent years owing to the high-throughput screening. However, more than 2/3 of these compounds exhibit low solubility in water, which dramatically decreases the potential of their usage and successful transfer to pharmaceutical practice [1–3]. Hence, the increase in the absorption of water-insoluble drugs into the human circulatory system is the key challenge in pharmaceutical research and industry [4, 5]. One of the most commonly used and most promising ways in this regard is the utilization of amorphous solid dispersions [6]. In this type of dosage form, the drug is dispersed in a polymeric matrix (either molecularly or in the amorphous state), which leads to its increased aqueous solubility, dissolution, and bioavailability [7, 8].

One of the most commonly used polymers in pharmaceutical dosage forms is hypromellose (HPMC). It is a hydrophilic amorphous polymer with the glass transition temperature (T_g_) occurring in the 160–210°C temperature range and the thermal decomposition being reported in the 200–250°C range [9]. Since the nowadays trends in the pharmaceutical industry favor hot melt extrusion (HME) over other formulation techniques (due to being a solvent-free continuous operation process) [10–12], the overall stability of the solid dispersion at high temperatures above T_g_ is of key importance. Initially, the T_g_ of HPMC (and, by extension, the need for high temperature during HME) was lowered by the addition of various plasticizers, but this approach has often led to the crystallization of the mixed-in drugs [13]. Therefore, a modified HPMC with lowered T_g_ specifically designed for the HME processing was recently introduced under the name “Affinisol™ HPMC HME” [14] (from now on referred to only as “Affinisol”). Affinisol has significantly lower T_g_ of ~120°C and a correspondingly lower viscosity, which are both highly beneficial for the HME preparation of solid pharmaceutical dispersions [14, 15].

The popularity of Affinisol has led to its increased usage in pharmaceutical practice [16–40], which has, over time, extended outside of its initially intended low-T utilization. Nowadays, Affinisol is commonly attempted to be used at temperatures close to 200°C, either due to the high melting temperatures (T_m_) of the mixed-in drugs or other excipients or due to the consequent processing of the HME-prepared extrudate [15, 17, 23]. The latter is particularly relevant with the recent boom of the 3D-printing technique usage [41–45] in the field of pharmacy. Since the acceptable adhesion of the 3D-printed layers is achieved at relatively high temperatures (usually close to 190–200°C for Affinisol) [23], the thermal degradation of Affinisol has recently emerged as a pressing issue. Surprisingly, the literature provides little to no information on this matter. The official information on the Affinisol material published by the DuPont Company (Affinisol manufacturer) recommends the lowest and highest ranges of the neat polymer processing temperatures to be 135–155°C and 190–200°C, respectively, depending on the average molecular weight [15]. The latter temperature range should prevent/minimize the change of the polymer color; otherwise, Affinisol should be stable against thermal degradation at least up to 250°C. Gupta *et al.* [17] report for Affinisol a rapid loss of mass above 240°C and also a small step-wise mass loss at ~190°C. Apart from this information being somewhat contradictory, all available literature data on the thermal degradation of Affinisol were obtained under an inert N_2_ atmosphere, which is not consistent with the common pharmaceutical practice, where both the hot melt extrusion and 3D-printing are performed in the ambient atmosphere (air; laboratory humidity).

The goal of the present paper is to perform an extensive degradation study of the Affinisol™ HPMC HME material in both N_2_ and (most importantly) air atmospheres. The main body of the experimental work will be realized using differential scanning calorimetry (DSC) and thermogravimetric analysis (TGA) to fully cover the whole spectrum of relevant signals, i.e., the heat release/consumption and mass gain/loss associated with the Affinisol degradation. In addition, Raman microscopy, infrared spectroscopy, and optical microscopy will be used to characterize different grades of the degraded HPMC. The DSC and TGA data will be used to evaluate the non-isothermal degradation kinetics. Consequently, the kinetic predictions will be made to determine the safe temperature-time profiles for the HME and 3D printing of Affinisol.

## Experimental

The hypromellose under the tradename Affinisol™ HPMC HME 15LV (DuPont, USA) was measured as-purchased in its powder form. In addition, the Affinisol powder was also used as source material for a series of hot melt extrusion (HME) filament preparations. The HME process was performed using the single-screw extruder Noztek Touch (Noztek, Great Britain) equipped with a cylindrical brass nozzle (output diameter 1.75 mm). The series of extrusions were performed for temperatures T_e_ = 120–230°C (with the step of 10°C) with the screw rotation speed set to 25 rpm. This resulted in the Affinisol powder spending approx. 5 min in the heated zone of the extruder barrel. In this way, Affinisol HME filaments with a length of 20 cm were prepared for each T_e_.

The thermogravimetric data were obtained by using the STA (TGA) 449 F5 Jupiter instrument (Netzsch) equipped with a DSC/TG holder. The STA cell was purged with 50 ml·min^−1^ of either N_2_ or dry synthetic air. The measurements were performed for the as-purchased Affinisol placed in open low-mass Al pans; the sample masses were approx. 3 mg (accurately weighted to 0.01 mg). The thermal degradation measurements were realized as non-isothermal heating scans - performed in the 30–500°C temperature range at rates q^+^ = 0.5, 1, 2, 3, 5, 7, 10, and 20°C·min^−1^ (for the degradation in N_2_ atmosphere, additional measurement at 30°C·min^−1^ was performed).

The calorimetric data were measured using a heat flow differential scanning calorimeter DSC Q2000 (TA Instruments) equipped with an autosampler, RCS90 cooling accessory, and T-zero technology. The DSC was calibrated using the In, Zn, and Ga metal standards. The Affinisol samples were measured in either hermetically sealed low-mass Al pans (intrinsic air atmosphere) or in open low-mass Al pans under the inert N_2_ atmosphere in the DSC cell. The sample masses were approx. 3 mg (accurately weighted to 0.01 mg). The DSC experiments were for the Affinisol powder realized as a series of heating scans at 0.5, 1, 2, 3, 5, 7, 10, 15, 20, 30, and 50°C·min^−1^ in the 10–300°C temperature range. In addition, a series of DSC supplemental characterization measurements were performed for the Affinisol filaments extruded at different temperatures – the DSC measurements were performed in hermetically sealed Al pans (air atmosphere) at q^+^ = 20°C·min^−1^ in the 10–300°C temperature range. Lastly, a supplemental series of degradation kinetics measurements were performed for the Affinisol filament extruded at 120°C; the parameters of the DSC experiments were: air atmosphere; 10–300°C range; q^+^ = 0.5, 1, 2, 3, 5, 7, 10, 15 20 and 30°C·min^−1^.

The structural characterization of the extruded Affinisol filaments and typical stages of the Affinisol powder degradation was done by means of Raman and infrared spectroscopy. The Raman spectra were obtained using the DXR2 Raman microscope (Nicolet, Thermo Fisher Scientific) equipped with the 785 nm excitation diode laser (30 mW, laser spot size 3.1 μm) and CCD detector. The experimental settings for the Raman measurements were 20 mW laser power on the sample, 1 s duration of a single scan, and 100 scans summed in one spectrum. The infrared spectra were obtained using the Fourier-transform infrared (FTIR) spectrometer Nicolet 6700 (Thermo Fisher Scientific) using a KBr pellet technique (the samples were homogenized with KBr); each FTIR spectrum was obtained by accumulating 128 scans at 1 cm^−1^ resolution. The morphology of the extruded Affinisol filaments and partially degraded Affinisol powders was investigated using two optical microscopes: a calibrated Dino-Lite Edge 3.0 adjustable microscopic probe and the iScope PLMi (Euromex) optical microscope equipped with ×10 and ×20 high-quality objectives and Moticam visual camera.

## Results

The present section will be split into two parts. In the first part, the TGA and DSC data for the Affinisol powder will be compared at different q^+^ and reaction atmospheres (N_2_ and O_2_). In addition, the DSC data obtained for the HME filaments will also be presented, showing the gradual influence of the extrusion temperature T_e_. In the second part, the Raman and infrared spectra will be shown for various stages of the decomposition process of both, Affinisol powder and HME filaments. The optical micrographs documenting the morphological changes of both types of samples will also be introduced.

### TGA and DSC Data

**Fig. 1:**
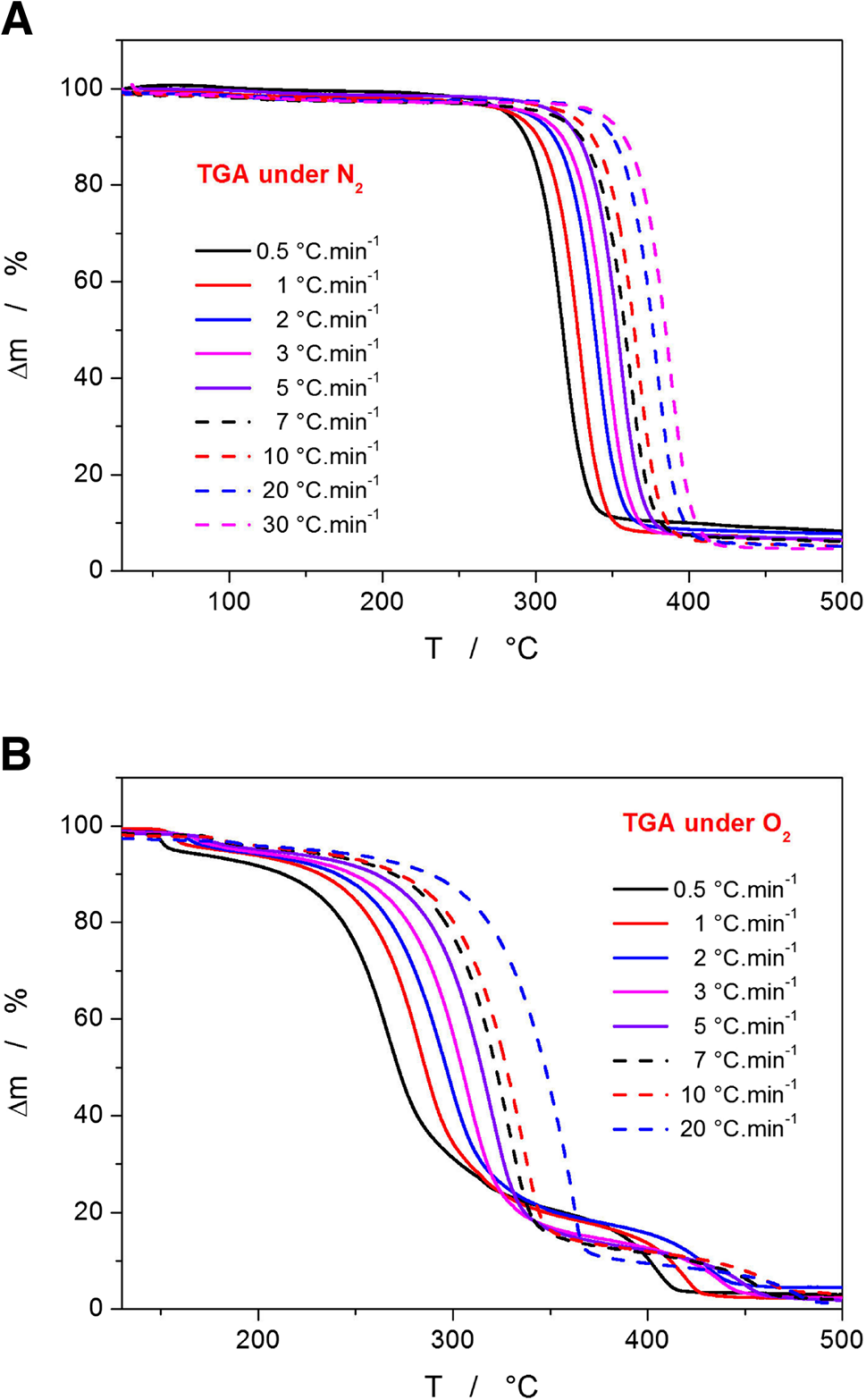
**(A)** TGA data obtained for the decomposition of Affinisol powder in N_2_ atmosphere at different q^+^. (**B)** TGA data obtained for the decomposition of Affinisol powder in an air atmosphere at different q^+^.

The standard record of the Affinisol thermal degradation under the N_2_ atmosphere is depicted in Fig. 1A. These data are consistent with the majority of literature reports [15, 17, 20] – i.e., with the degradation proceeding in a single step and with Affinisol showing good thermal stability up to ~250°C even at very low q^+^ (the mass loss onset determined under such conditions usually exhibits a similar degree of degradation to the short isothermal annealings, such as those encountered during the real-life extrusion or 3D printing processes). At the highest q^+^ = 30°C·min^−1^, evoking the situations when the biopolymer very shortly overheats during the local temperature fluctuations, the Affinisol starts to slowly decompose at ~320°C. The overall mass loss ranges from approx. 89 to 95%. However, in pharmaceutical practice (both laboratory and industrial), Affinisol is very rarely processed under an inert atmosphere. The absolute majority of the high-T operations involving Affinisol proceeds in air, with O_2_ having direct access to the heated material.

The TGA data measured in the air atmosphere are shown in Fig. 1B. As is apparent, this degradation profile is completely different, exhibiting a three-step mass loss in the observed temperature range. The first small loss of sample mass is initiated in the 135°C (at 0.5°C·min^−1^) – 180°C (at 20°C·min^−1^) temperature range. Interestingly, the mass loss percentage decreases with the heating rate from 5 to 1% in the explored q^+^ range, which indicates that the rate of this reaction is slow and diffusion-controlled. In addition, no alternative for this reaction step occurs under the N_2_ atmosphere, which infers direct involvement of the O_2_ from the air. The first mass loss step is practically immediately followed by a slowly initiated second/dominant mass loss, which proceeds in a roughly similar temperature range (250–375°C) as the single-step mass loss occurring under the N_2_ atmosphere (260–420°C). The kinetics of the second decomposition step is not consistent over the whole range of applied q^+^ − a marked change occurs between the 0.5–2°C·min^−1^ and 3–20°C·min^−1^ datasets, which indicates a competing reaction mechanism. Note that the TGA curve obtained at 2°C·min^−1^ already exhibits signs of an intermediate behavior between the two reaction routes.

In particular, at low q^+^, the no. 1 reaction route products form in large amounts already below ~310°C, and the mass loss in the second reaction step accounts for approx. 75% of the overall sample mass. In this case, the consequent third reaction step represents the mass loss of ~18% and gradually follows/interlinks with the second reaction step – this is a strong indication of the third reaction step being limited rather by the availability of the intermediate reaction products (from the second reaction step) than by temperature/energy barrier for the reaction activation. On the other hand, the second reaction route manifesting at q^+^ ≥ 3°C·min^−1^ results in the second mass loss proceeding more steeply and accounting for approx. 80% of the overall mass. The consequent third reaction step is then separated by a significantly larger temperature gap, and the mass loss corresponding to the third step is ~13%.

**Fig. 2:**
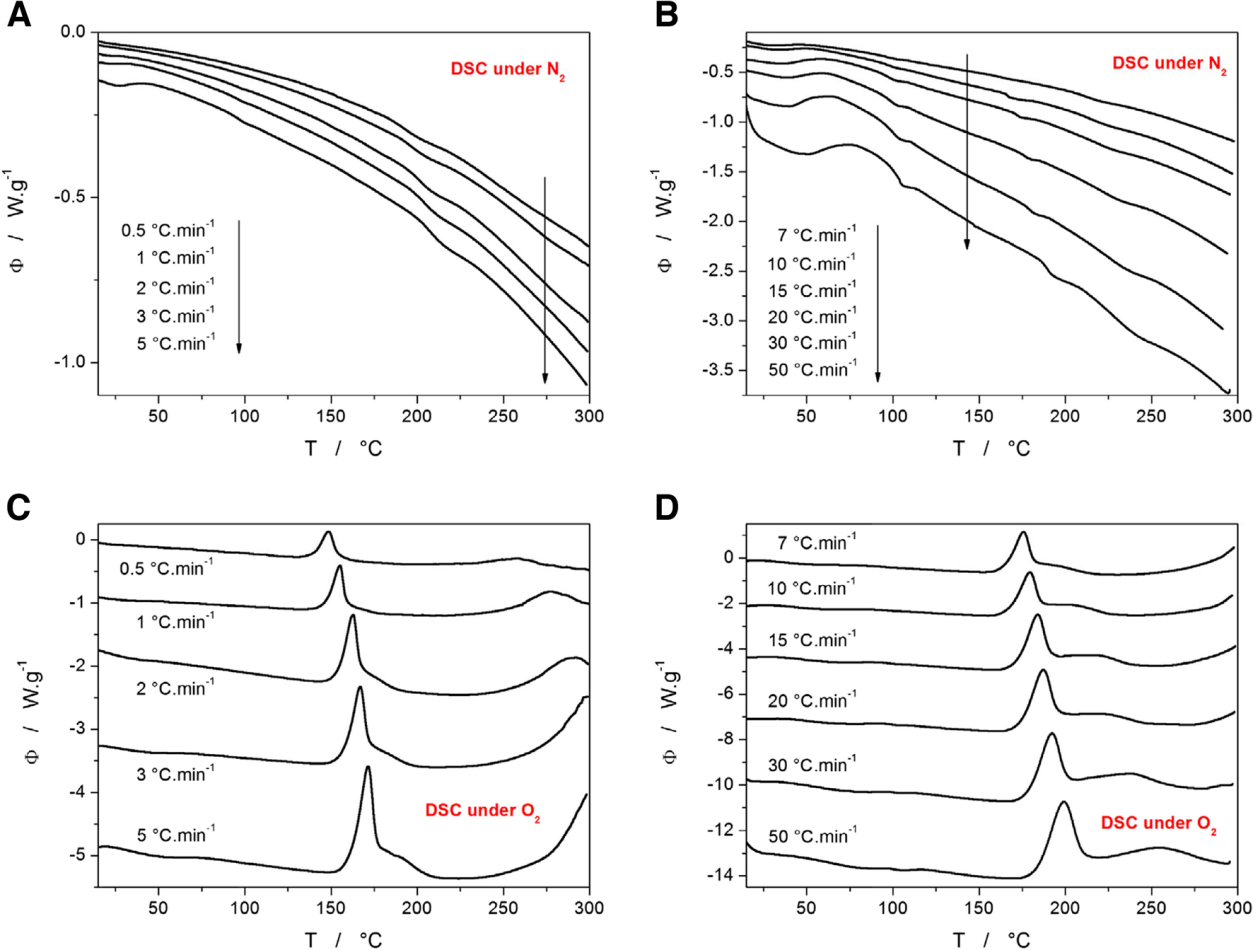
**(A)** DSC data obtained for the low-temperature decomposition of Affinisol powder in N_2_ atmosphere at low q^+^. (**B)** DSC data obtained for the low-temperature decomposition of Affinisol powder in an N_2_ atmosphere at high q^+^. (**C)** DSC data obtained for the low-temperature decomposition of Affinisol powder in an air atmosphere at low q^+^. (**D)** DSC data obtained for the low-temperature decomposition of Affinisol powder in an air atmosphere at high q^+^. Exothermic effects in all graphs evolve in the upwards direction.

The complementary DSC data monitoring the primary stage of the Affinisol degradation in the 20–300°C temperature range are shown in Fig. 2. In the case of the measurements performed under the N_2_ atmosphere (see Fig. 2A and B), the DSC curves first show a shallow wide endothermic dip in the 30–70°C temperature range (corresponding to the release of physically adsorbed water). Another effect is the endothermic step change accompanied by a small endothermic peak at ~110°C (corresponding to the glass transition and structural relaxation peak of the amorphous polymer) [15–20]. At higher temperatures, two additional small endothermic effects can be observed near 180 and 230°C – considering the small magnitude and thermal tone of these signals, they are most probably associated with minor thermally initiated/forced structural changes of Affinisol. Note that none of these effects is associated with mass loss.

A strikingly different situation arises for the DSC measurements performed in the air atmosphere – see Figs. 2C and 2D. Here, three strong exothermic peaks occur, associated with spontaneous oxidation-based chemical reactions that are directly associated with the loss of mass. The first strong exothermic signal occurs at 135°C at 0.5°C·min^−1^, and its onset gradually shifts with q^+^ to 175°C (for 20°C·min^−1^). The increasing heating rate also leads to the formation of an increasingly separated high-T shoulder of the first exothermic peak that eventually segregates into a second individual exothermic peak (manifesting in the 220–290°C range at 50°C·min^−1^). The third recognizable exothermic signal that corresponds to the main decomposition mass loss occurs in the DSC data in the 200–300°C range at 0.5°C·min^−1^. With increased q^+^, this signal shifts in accordance with the TGA data (see Fig. 1B) to higher temperatures, and for q^+^ ≥ 30–50°C·min^−1^, the onset of the main decomposition process shifts outside of the DSC-measured temperature range (i.e., above 300°C).

**Fig. 3:**
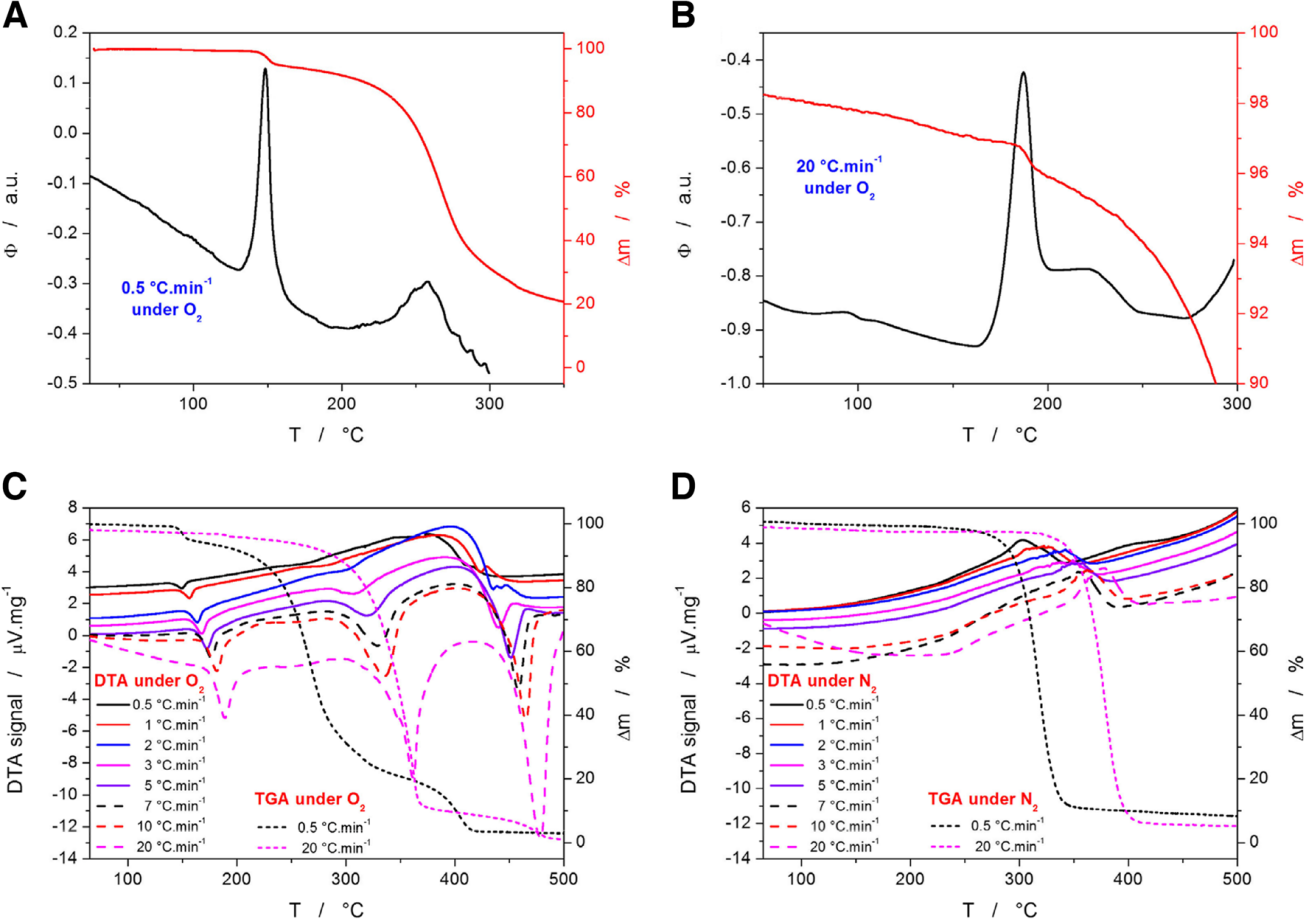
**(A)** Comparison of the TGA and DSC curves obtained at 0.5°C·min^−1^ in air atmosphere. (**B)** Comparison of the TGA and DSC curves obtained at 20°C·min^−1^ in air atmosphere. (**C)** DTA data obtained for the decomposition of Affinisol powder in an air atmosphere at different q^+^. The right axis and the dotted data show the TGA curves obtained at 0.5°C·min^−1^ 20°C·min^−1^ in air atmosphere. (**D)** DTA data obtained for the decomposition of Affinisol powder in an N_2_ atmosphere at different q^+^. The right axis and the dotted data show the TGA curves obtained at 0.5°C·min^−1^ 20°C·min^−1^ in the N_2_ atmosphere. Exothermic effects in graphs A and B evolve in the upwards direction; for graphs C and D, the exothermic effects evolve in the downward direction.

A direct comparison of the TGA and DSC data is demonstrated in Fig. 3A and B. The data obtained at 0.5°C·min^−1^ display the marked disproportion between the enthalpy and mass loss manifestations of the degradation processes. Whereas the first and second mass decreases are in a ~ 1/15 ratio, the corresponding enthalpy changes (represented by the merged first+second *vs*. third DSC peaks) are roughly similar in magnitude. This indeed confirms the strong oxidative nature of the first mass loss effect at ~140°C, where the evolved heat per released mass is very high compared to the second (main) mass loss effect. The measurement performed at 20°C·min^−1^ (Fig. 3B) shows that the initial small mass loss effect is associated solely with the first (sharp) DSC peak and not with its high-T shoulder (denoted as the second DSC peak in the previous text). Close examination of the TGA curve in the 150–180°C temperature range reveals a slight relative increase of mass preceding the first mass loss effect (note the term “relative”, indicating that the effect is overlapped by the continuous underlying decrease of the sample mass) – similar effects are present for all q^+^. This indicates that the oxidation reaction proceeds at least initially in the solid-state form. Hence the slight relative increase in the mass.

The comparison of the TGA and DTA (measured simultaneously by the STA instrument) data in the full temperature range is shown in Fig. 3C and D for the air and N_2_ atmospheres, respectively. For better clarity, all DTA records are displayed, but they are compared only with the TGA data curves obtained at the lowest and highest q^+^. In the case when the material had access to O_2_, all mass loss effects are accompanied by relatively strong exothermic (oxidative) signals. For the second (main) TGA mass decrease, the exothermic signal is weak at low q^+^ and markedly increases for q^+^ ≥ 3°C·min^−1^. This is further evidence of the competitive decomposition reactions being involved, where one appears to be associated with the stronger oxidation effect – as also discussed previously in Fig. 1B. It is also noteworthy that at higher q^+^, the amount of released heat increases with each consequent mass loss step, indicating that more products prone to the oxidation are generated in disproportion with the actual mass being lost. The measurements performed under the N_2_ atmosphere exhibit only a single mass loss associated with an endothermic DTA peak, which is consistent with the typical temperature-induced decomposition behavior.

**Fig. 4:**
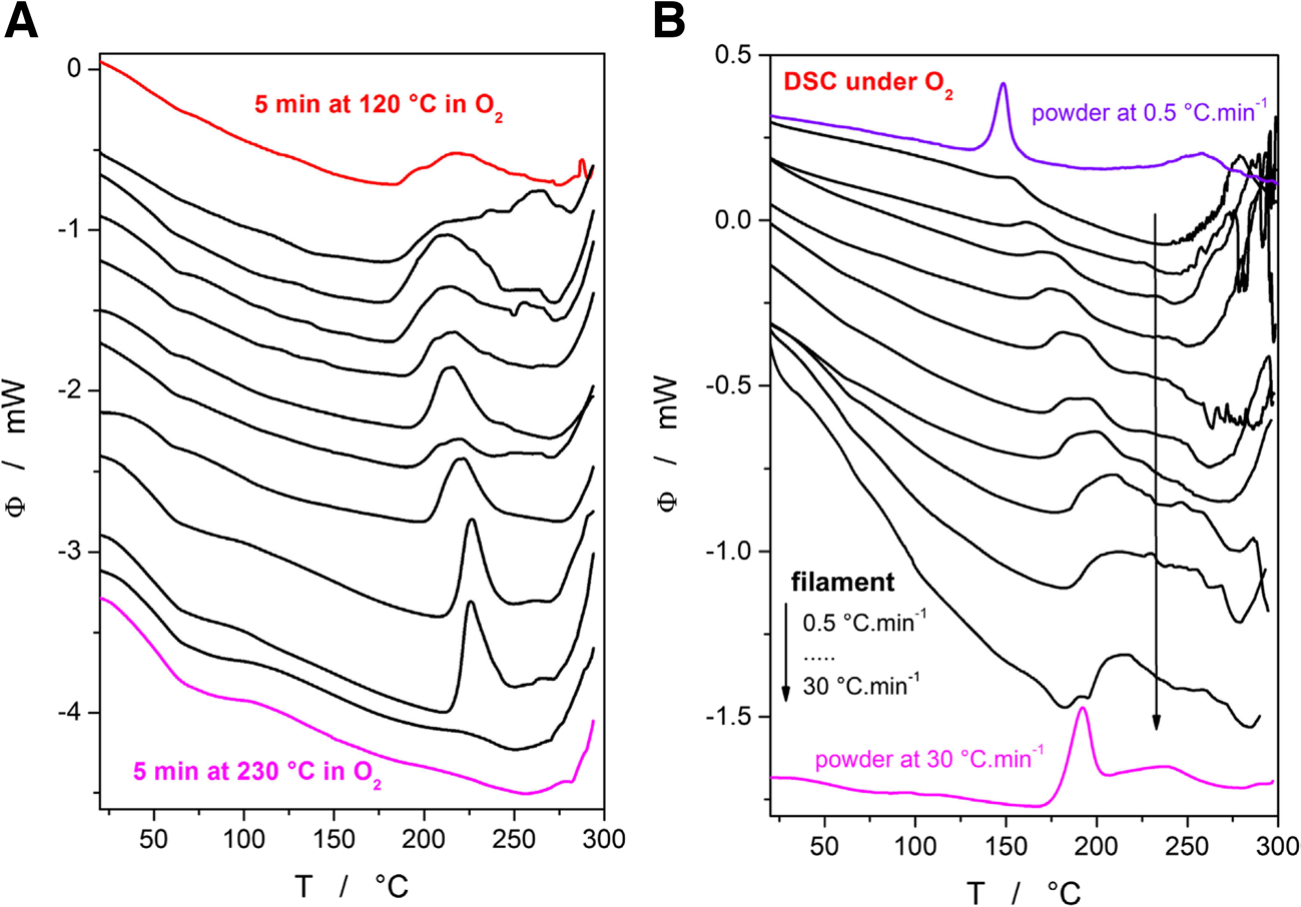
**(A)** Series of DSC measurements performed (at 20°C·min^−1^ in the air) for the Affinisol filaments extruded at different T_e_ temperatures – from 120 to 230°C with a step of 10°C. The curves are aligned from top to bottom according to increasing T_e_; the curves measured for the lowest and highest T_e_s are indicated. (**B)** The DSC curves obtained in the air at different q^+^ for the Affinisol filament extruded at T_e_ = 120°C. Two examples of the akin DSC measurements performed for the Affinisol powder are indicated in color. Exothermic effects in all graphs evolve in the upwards direction.

In addition to the Affinisol powder, the Affinisol filaments HME-produced at different temperatures (from 120 to 230°C with the step of 10°C; the retention time of the material in the heated zone of the extruder was estimated to be 5 min) were also measured by DSC to reveal the potential changes in their thermo-kinetic behavior. The corresponding DSC curves measured at 20°C·min^−1^ under air atmosphere are displayed in Fig. 4A. As is apparent, the extrusion at temperatures up to ~180°C results only in minimum changes of the joined first+second DSC peaks (associated with the first small mass loss). At higher extrusion temperatures (210°C ≥ T_e_ ≥ 190°C), the exothermic peak shifts to higher temperatures and gets progressively sharper, which indicates that the oxidative decomposition reaction has already (to a very small extent) proceeded during the hot melt extrusion process and that the diversity of the reaction centers has decreased (the scarce active spots with lower activation energetic barriers have already reacted). For the extrusion temperatures T_e_ ≥ 220°C, the first oxidative decomposition has already fully proceeded during the HME procedure.

In addition to the measurements presented in Fig. 4A, a series of standard kinetic measurements in the air atmosphere was performed for the Affinisol filament extruded at 120°C (where no degradation is expected to happen yet). The aim was to explore the impact of the fused-together compact filament form on the diffusion of O_2_ into the bulk material. The data are displayed in Fig. 4B, together with the selected measurements performed on the Affinisol powder. The measurements performed on the HME-processed Affinisol are shifted relatively insignificantly to higher T (onsets by approx. 5–10°C, peaks by approx. 20°C). The shape of the DSC degradation peaks is also more diffused and elongated - most probably due to the gradually delayed access of O_2_ inwards the Affinisol material. The scattered DSC peaks corresponding to the measurements of HME-processed filaments at higher q^+^ testify about the macroscopically heterogeneous structure of the filaments, where the oxidation is severely limited by the O_2_ access to the partially decomposing material (see Fig. 3B).

### Microscopic and Spectroscopic Data

**Fig. 5:**
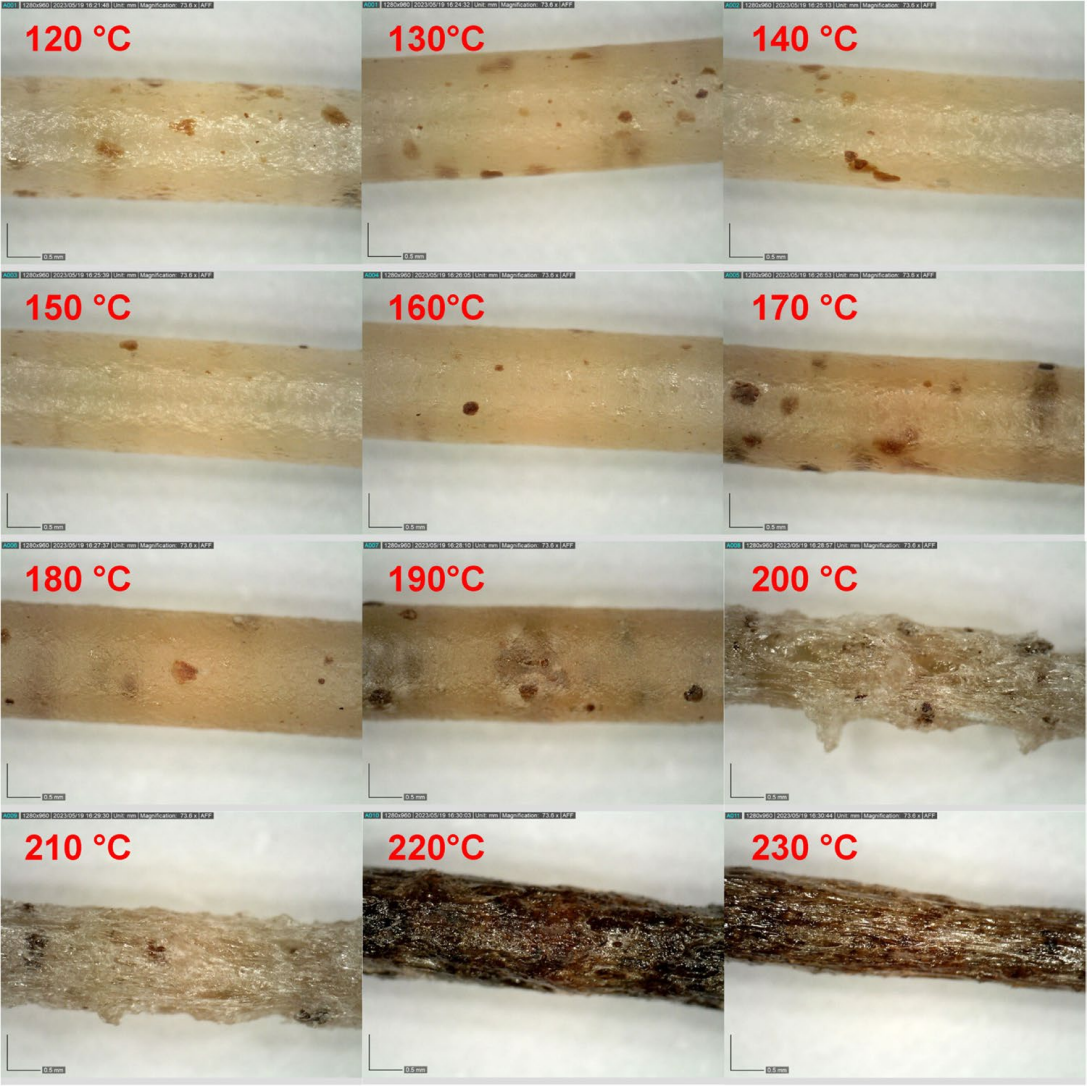
Micrographs of the Affinisol filaments extruded at different T_e_s (indicated in each micrograph). The scale bars correspond to 0.5 mm.

Optical microscopy was used to judge the shape and texture of the Affinisol filaments extruded at various temperatures – see Fig. 5. Up to the extrusion temperature of approx. 160°C, the surface texture, thickness, and color of the filament remained practically unchanged. In the 170–190°C range, the filaments have become slightly darker, with a more frequent occurrence of dark brown spots; also, the surface of the filaments has become dimmer and opaquer. In the 200–210°C temperature range, the filaments become lacerated with a strongly fissured surface. At even higher temperatures, 220 and 230°C, the filaments have a deep brown color, indicating a severe burning/oxidation of the decomposing material – these processes also corrupt the continuity of the extrusion procedure, leading to the non-uniform filament diameter.

**Fig. 6:**
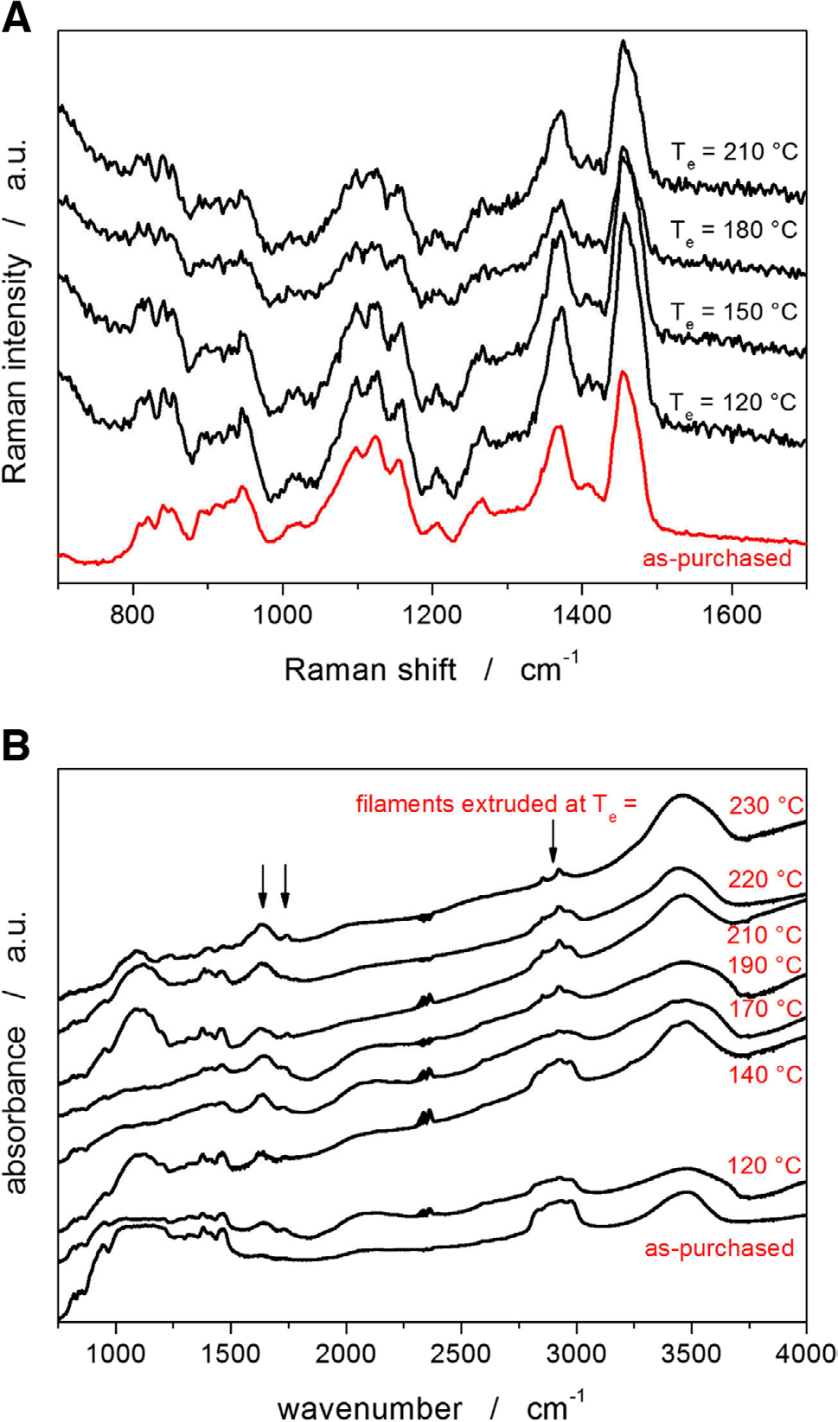
**(A)** Raman spectra for the as-purchased powder Affinisol and for the Affinisol filaments extruded at selected temperatures. (**B)** FTIR spectra for the as-purchased powder Affinisol and for the Affinisol filaments extruded at selected temperatures. Arrows indicate the discussed bands.

Apart from optical microscopy, Raman microscopy, and infrared spectroscopy were used to investigate the structural changes in Affinisol caused by the high-T extrusion. Pyrolysis of the cellulose and HPMC (see the inset of Fig. 6A for the molecular structure of HPMC) was described in [15–17, 19, 20, 46–50] based on the GC-MS (gas chromatography paired with mass spectrometry), thermogravimetry, as well as DFT (density functional theory) data. Practically all papers report on the thermal decomposition in N_2_, introducing numerous reaction pathways, most of which are true to some extent observed in real experiments. The complexity of the decomposition process can be expressed by the following findings: 1) The decomposition of HPMC was in [12] monitored by means of joint TGA and gas analyzer, and in the 200–300°C temperature range, the H_2_O, CO, CO_2_, CH_4_, C_2_H_6_, and C_3_H_8_ were detected. Pyrolysis of cellulose in the 430–730°C range was studied in [49] by means of joint TGA and FTIR (Fourier-transformation infrared spectrometer); 26 reaction pathways involving over 45 reactions and over 50 intermediary/final reaction products were suggested. The initial pyrolysis mechanism of cellulose and cellobiose (basic structural unit of cellulose, with glucose monomers and glycosidic bonds) was studied by paired gas chromatography and mass spectrometry in [47] – 11 reaction pathways delinearizing the cellulose polymeric chain were suggested in the 400–600°C range (30 compounds were identified as the main reaction products of the rapid pyrolysis). The absolute majority of the pyrolysis HPMC products can be divided [47, 49, 50] into three groups: pyrans (levoglucosan), furans (5-hydroxyfurfural, furfural), and linear small molecule compounds (H_2_O, CO, CO_2_, acetaldehyde, glycolaldehyde, acetic acid …). Regarding the low-T decomposition, the gas analysis reported [12] mainly H_2_O and low amounts of CO being released during degradation at 200°C in air. Considering the great complexity of the HPMC decomposition mechanism, only binary information (yes/no) about the possible alterations of the Raman and FTIR spectra was sought in the present study (rather than identification of the particular reaction mechanisms involved).

The Raman spectra corresponding to the Affinisol filaments HME-processed at selected temperatures are shown in Fig. 6A. As is apparent, the Raman spectra do not show significant changes with the extrusion temperature T_e_ (note that at T_e_ ≥ 220°C, the Raman spectra exhibited very strong fluorescence, indicating high content of solid-state defects or traces of transition metal ions [51]. The similarity of the Raman spectra indicates that the heat-induced partial (between 2 and 10 w.% mass loss) oxidative decomposition of Affinisol has either very little solid-state reaction residue (note the relatively low sensitivity of the Raman spectroscopy) or these have no active Raman vibrations.

Therefore, infrared spectroscopy was used to gain deeper insight into the potential structural changes associated with the first decomposition step of Affinisol heated in the air atmosphere. The FTIR spectra of the HME-processed Affinisol filaments are shown in Fig. 6B (note the processing of the spectra that involved the subtraction of the signals corresponding to H_2_O and CO_2_ contained in the air – hence the scattered signal at 2350 cm^−1^). The assignment of the major Affinisol FTIR bands can be done as follows [46, 48, 49]: 800–850 cm^−1^ band corresponds to the rocking mode of CH_2_, 950–1000 cm^−1^ band corresponds to the asymmetric stretching mode of pyranose, 1000–1100 cm^−1^ band corresponds to the stretching mode of ethereal C-O-C groups, 1250–1300 cm^−1^ band corresponds to the symmetric vibrations of cyclic epoxide C-O-C groups, 1350–1400 cm^−1^ band corresponds to the symmetric vibrations of cyclic anhydrides C-O-C groups, 1450–1500 cm^−1^ band corresponds to the asymmetric bending vibrations of methoxy group, 1600–1650 cm^−1^ band corresponds to the stretching vibrations of C-O in six-membered cyclic rings, 1680–1780 cm^−1^ band corresponds to the stretching vibrations of C=O aldehydes, ketones, and carboxylic acids, 2900 cm^−1^ band corresponds to the symmetric stretching vibrations of methyl and hydroxypropyl groups, 3400–3500 cm^−1^ band corresponds to the stretching vibrations of OH groups.

Based on the data depicted in Fig. 6B, the increase of the extrusion temperature T_e_ above 170°C leads to an abrupt relative decrease in the intensity of the group of bands at 2800–3000 cm^−1^, which correspond to the vibrations of methyl and hydroxypropyl units [46]. This may suggest a partial split of these units from the main cellulose polymeric backbone during the initial decomposition step (with the adjacent reaction products being methanol and 1,2-propanediol). Thermal processing of Affinisol also appears to result in the relative increase of the bands at 1630 cm^−1^, which may indicate the gradually increasing formation of levoglucosan [49, 50] (product of one of the main reaction pathways of the initial cellulose degradation). Similarly, increasing the band at 1740 cm^−1^ then indicates the formation of aldehydes and ketones, which represent the main reaction products of the alternative reaction pathways for the initial cellulose degradation [49, 50]. As was reported in [12], mainly H_2_O and small amounts of CO are released into the gas phase during degradation near 200°C, which is consistent with the reaction mechanisms from [49, 50]. Oxidation of the released CO could then represent one of the main exothermic sources detected by DSC (see Fig. 2D). The further degradation of Affinisol that proceeds at higher temperatures (associated with the second and third mass loss steps) is expected to proceed similarly to that of pure cellulose [49] – even more so if the first mass loss in air indeed corresponds to the loss of the methyl and hydroxypropyl groups, which would leave the cellulose backbone.

## Discussion

The present section will be split into two parts: First, the DSC and TGA data introduced in Section 3.1. will be described in terms of the standard solid-state kinetic models, and a discussion will be conducted regarding the mutual inter-relationships of the particular steps within the overall reaction mechanism. In the second part, the theoretically simulated kinetic predictions will be calculated based on the kinetic description introduced in Section 4.1. The simulated kinetic behavior (extrapolated outside of the experimentally measured data) will be utilized to discuss the optimum HME and 3D printing temperatures, and to determine the main limitations for the thermal processing of Affinisol HPLC HME. Note that the nowadays standard processing temperatures of raw Affinisol are ~160–170°C for HME and ~ 200–210°C for 3DP [23].

### Decomposition Kinetics

The decomposition kinetics is traditionally described in terms of the basic solid-state kinetics expression [52] (Eq. 1) relating the conversion rate between reactants and products to either heat flow changes (in calorimetric techniques; Eq. 2) or mass changes (in thermogravimetric techniques; Eq. 3):

1 $$da/ dt=A\bullet {e}^{-E/ RT}\bullet f(a)$$

2 $$da/ dt=\Phi /\Delta H$$

3 $$da/ dt=d\Delta m/ dt$$

where dα/dt is the rate of conversion (with α being the degree of conversion and t being time), A is the pre-exponential factor, E is the apparent activation energy of the decomposition process, R is the universal gas constant, T is temperature, f(α) is the mathematical function for the given kinetic model, *Φ* is the calorimetric heat flow, Δ*H* is the enthalpy change associated with the decomposition/degradation, Δm is the normalized sample mass in percent. Regarding the appropriate kinetic model, a large list of both physically meaningful and empirical solid-state models can be found in [53]. Considering the large variety of the kinetic signal asymmetries (see, e.g., Figs. 1 and 2C, D) , the flexible empirical autocatalytic Šesták-Berggren model [54] (AC, Eq. 4) will be used in the present paper:

4 $$f{(a)}_{AC}={a}^M{\left(1-a\right)}^N$$

where M and N are the AC model kinetic exponents that can be interpreted as the extent of the autocatalysis and the reaction order, respectively.

The enumeration of Eqs. 1–4 can be done utilizing a number of linearization or non-linear optimization methods, as described, e.g., in [53]. In the present paper, the recently developed combination of the temperature-resolved activation energy fixation (determined using the Kissinger method [55] – see Eq. 5) and single-curve multivariate kinetic analysis sc-MKA method [56] (see Eqs. 6 and 7) will be applied:

5 $$1\textrm{n}\left(\frac{q^{+}}{T_p^2}\right)=-\frac{E}{RT_p}+ const.$$

6 $$RSS=\sum\limits_{j=1}^n\sum\limits_{k= Firs{t}_{j^i}}^{Last_j}{w}_{j,k}{\left(Y\ \textrm{ex}{\textrm{p}}_{j,k}- Yca{l}_{j,k}\right)}^2$$

7 $${w}_j=\frac{1}{{\left|{\left[ da/ dt\right]}_{\textrm{max}}\right|}_j+{\left|{\left[ da/ dt\right]}_{\textrm{min}}\right|}_j}$$

where T_p_ is the temperature corresponding to the maximum of the decomposition peak or to the maximum decomposition rate (inflection) in the case of the TGA data; *RSS* is the sum of squared residue, *n* is the number of measurements, *j* is an index of the given measurement, *First*_*j*_ is the index of the first point of the given curve, *Last*_*j*_ is the index of the last point of the given curve, *Yexp*_*j,k*_ is the experimental value of the point *k* of curve *j*, *Ycal*_*j,k*_ is the calculated value of the point *k* of curve *j*, and *w*_*j*_ is a weighting factor for curve *j*. The Y_cal_ is calculated on the basis of Eqs. 1–4.

**Fig. 7:**
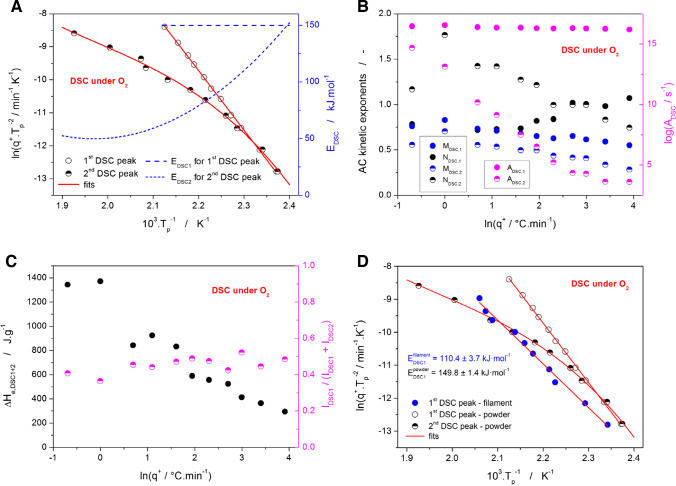
**(A)** Kissinger dependences for the two overlapping DSC peaks (see Fig. 2C and D) measured for Affinisol powder in the air. The overlap corresponds to the initial/first decomposition step. The right axis and the dashed + dotted lines show the temperature evolution of activation energy for the two overlapping peaks/processes determined from the derived Kissinger dependences. (**B)** Kinetic parameters A, M, and N (determined by the sc-MKA method with E fixed on the values shown in Fig. 7A) for the two overlapping DSC peaks measured for the Affinisol powder in air plotted in dependence on q^+^. (**C)** The overall enthalpy (sum for the two overlapping DSC peaks corresponding to the initial decomposition stage; see Fig. 2C and D) and the portion of the area corresponding to the first/dominant DSC peak measured for the Affinisol powder in the air. The data are plotted in dependence on q^+^. (**D)** Comparison of the Kissinger dependences shown in Fig. 7A with the Kissinger dependence constructed for the DSC measurements performed in the air for the Affinisol filament extruded at 120°C.

Starting with the DSC data for the initial decomposition step in the air atmosphere (see Fig. 2C and D), the kinetic peak is complex and strongly exothermic, consisting of the dominant rapid process followed by a slower high-T degradation sub-process that gets partially separated at high q^+^. Since the preliminary MKA fits have shown that the activation energy of the high-T sub-process is not constant, the Fraser-Suzuki mathematical deconvolution [57] was used to separate the two exothermic DSC peaks. The Fraser-Suzuki deconvolution is based on the equation:

8 $$y={a}_0\exp \left[-1\textrm{n}\ 2{\left[\frac{1\textrm{n}\left(1+2{a}_3\frac{x-{a}_1}{a_2}\right)}{a_3}\right]}^2\right]$$

where a_0_, a_1_, a_2_, and a_3_ are parameters responsible for the magnitude, position on the X axis, width, and asymmetry, respectively. In the present case, the Fraser-Suzuki mathematical deconvolution was performed only to obtain the T_p_ values of both overlapping exothermic peaks, to construct the Kissinger plot, and to determine the E-T dependences. The Kissinger plot and the corresponding evaluated activation energies are shown in Fig. 7A. Whereas the sharp/dominant decomposition peak shows perfectly linear Kissinger dependence (r^2^ = 0.9992; indicating the uniformity of the decomposition mechanism) with E_DSC1_ = 149.8 ± 1.4 kJ·mol^−1^, its high-T shoulder indeed exhibits strong dependence of E on T. This E-T dependence was calculated based on the derivation of a fit by 3^rd^-polynomial function. At high q^+^, E_DSC2_ ≈ 50 kJ·mol^−1^, and with decreasing q^+^, it converges to the data of the sharp/dominant decomposition peak. This, together with the visual convergence of the two peaks (as depicted in Fig. 2C and D), confirms that the high-T shoulder represents a consequent/following reaction, the reactants of which are the products of the preceding reaction step associated with the sharp/dominant decomposition peak.

As such, the reaction mechanism was represented by the following set of equations:

9 $$\frac{da}{dt}=-{A}_1\bullet \exp \left(-\frac{E_1}{RT}\right)\bullet {f}_1\left(a,b\right)$$

10 $$\frac{db}{dt}={A}_1\bullet \exp \left(-\frac{E_1}{RT}\right)\bullet {f}_1\left(a,b\right)-{A}_2\bullet \exp \left(-\frac{E_2}{RT}\right)\bullet {f}_2\left(b,c\right)$$

where “a” is the Affinisol polymer, “b” are the products of the first reaction step, and “c” are the products of the second reaction step – all expressed as the degree of conversion, either α or (1-α). The sc-MKA results based on the combination of Eqs. 4, 6, 7, 9, and 10 (and fixed E_DSC_ values from Fig. 7A) determined for each DSC data curve separately are shown in Fig. 7B and C as a dependence on q^+^. Whereas the asymmetry of the sharp/dominant degradation peak is fairly uniform at q^+^ ≤ 5°C·min^−1^ (indicated by constant M and N AC kinetic exponents), the shape of the consequent high-T process changes rather dramatically with q^+^. The constant ratio between the integrated areas corresponding to the two overlapping processes and the markedly decreasing overall enthalpy suggest that the first decomposition sub-process is driven by the diffusion of O_2_ into the Affinisol matrix, and the second sub-process corresponds to further oxidation of the formed products. The former sub-process appears to be the rate-determining reaction step.

In addition to the DSC measurements of powdered Affinisol, the small pieces of the Affinisol filament extruded at 120°C were also subjected to a similar series of kinetics measurements – see Fig. 4B. Taking into account the irregular shape of the DSC peaks (unsuitable for precise model-based curve-fitting by sc-MKA), only the Kissinger methodology was used to determine the decomposition activation energy. The corresponding Kissinger dependence is depicted in Fig. 7D together with the data for the powdered Affinisol (taken from Fig. 7A). The compact nature of the extruded samples clearly leads to a shift of the decomposition process to higher temperatures (by 5–15°C, depending on q^+^). Since thermal gradients within the samples are negligible, at least at lower q^+^, the shift is unambiguous evidence for the diffusion to be a rate-determining step for the oxidizing reaction. The shift to higher T also results in lower activation energy of ~110 kJ·mol^−1^, as is expected for the sterically restricted (on a macroscopic level) reaction/transformation. The pre-exponential factor and the AC model parameters corresponding to the Affinisol filament DSC data are: log(A/s^−1^) = 10.73, M = 0.63, N = 1.18.

**Fig. 8:**
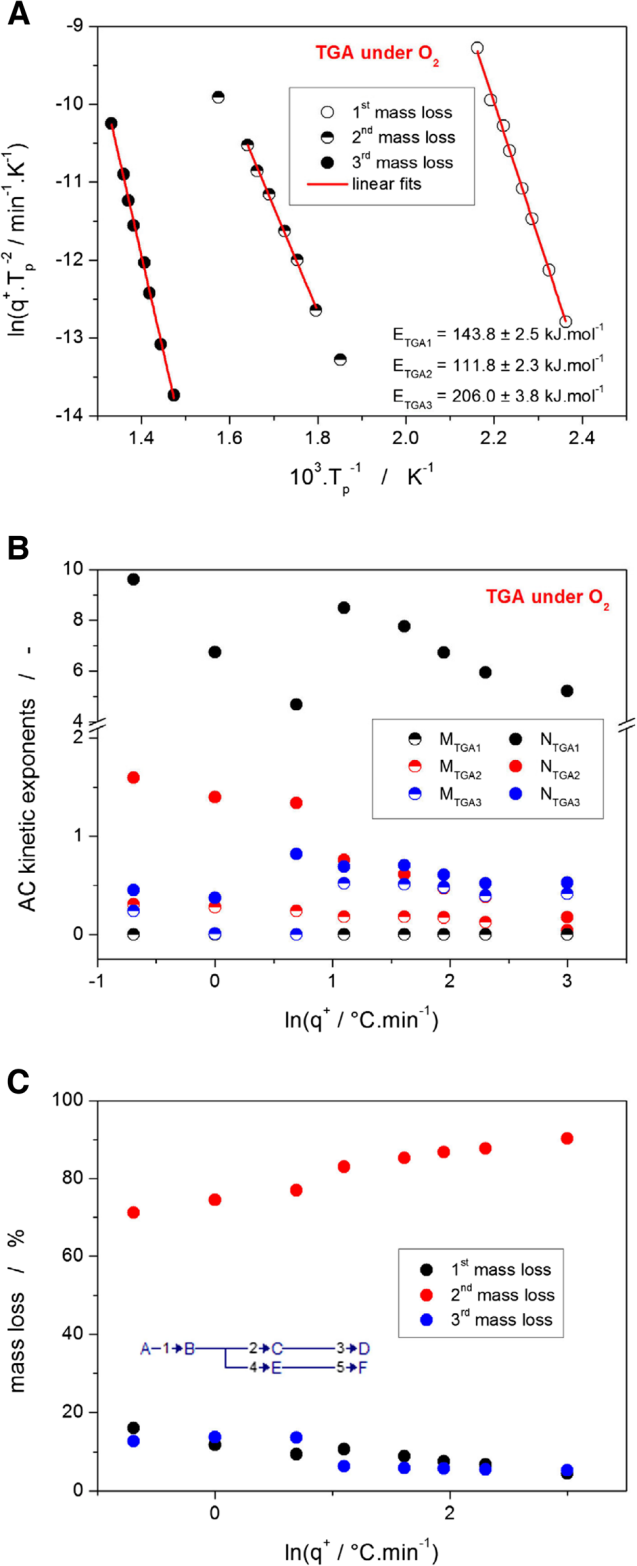
**(A)** Kissinger dependences for the three decomposition stages measured by TGA in the air – see Fig. 1B. (**B)** Kinetic parameters A, M, and N (determined by the sc-MKA method with E fixed on the values shown in Fig. 8A) for the three TGA decomposition stages measured for the Affinisol powder in the air. The data are plotted in dependence on q^+^. (**C)** Representation of the three particular TGA decomposition steps measured for the Affinisol powder in air as a percent portion of the overall mass loss. The depicted scheme represents the simplest reaction model that can be used to describe the TGA decomposition in air.

A similar sequence of the calculation procedures was also applied for the TGA data; the Fraser-Suzuki deconvolution was not used as a preliminary step because no full overlap of two mass loss steps occurred. Due to the large degree of potential reaction complexity associated with the existence of numerous reaction pathways and products (see the discussion on the spectroscopic data in Section 3.2.), all mass decreases were treated as independent reactions, and their interdependences will be discussed based on the trends in their kinetic description. The TGA data for the degradation of Affinisol in air atmosphere are shown in Fig. 8.

Whereas the Kissinger plots show very good linearity, indicating uniform activation energy for each decomposition step, the AC model parameters (corresponding to the peaks asymmetries) and the mass loss values for the three decomposition steps exhibit a marked discontinuity in-between the data-points corresponding to the 2 and 3°C·min^−1^. Since the shape of the first decomposition step was found to be possibly influenced by the onset of the second (largest) mass loss, the most probable simplest usable reactions scheme is that depicted in Fig. 8C: initial strongly oxidative diffusion-driven decomposition followed by the two competing reaction paths, where the split occurs at q^+^ > 2°C·min^−1^. Interestingly, the activation energy of the two reaction pathways appears to be very similar, which may suggest a mechanistic reason for the switch between the dominant reaction routes. It is worth noting that in the case of the second (main) mass loss, the first and last points (obtained at the lowest and highest q^+^) were omitted from the evaluation. The high-q^+^ point deviated probably due to the temperature gradients or delayed mass diffusion; the deviation of the low-q^+^ point was caused by the inaccurate determination of the inflection point on the TGA curve due to the overlap of the second and third mass losses (see Fig. 1B).

**Fig. 9:**
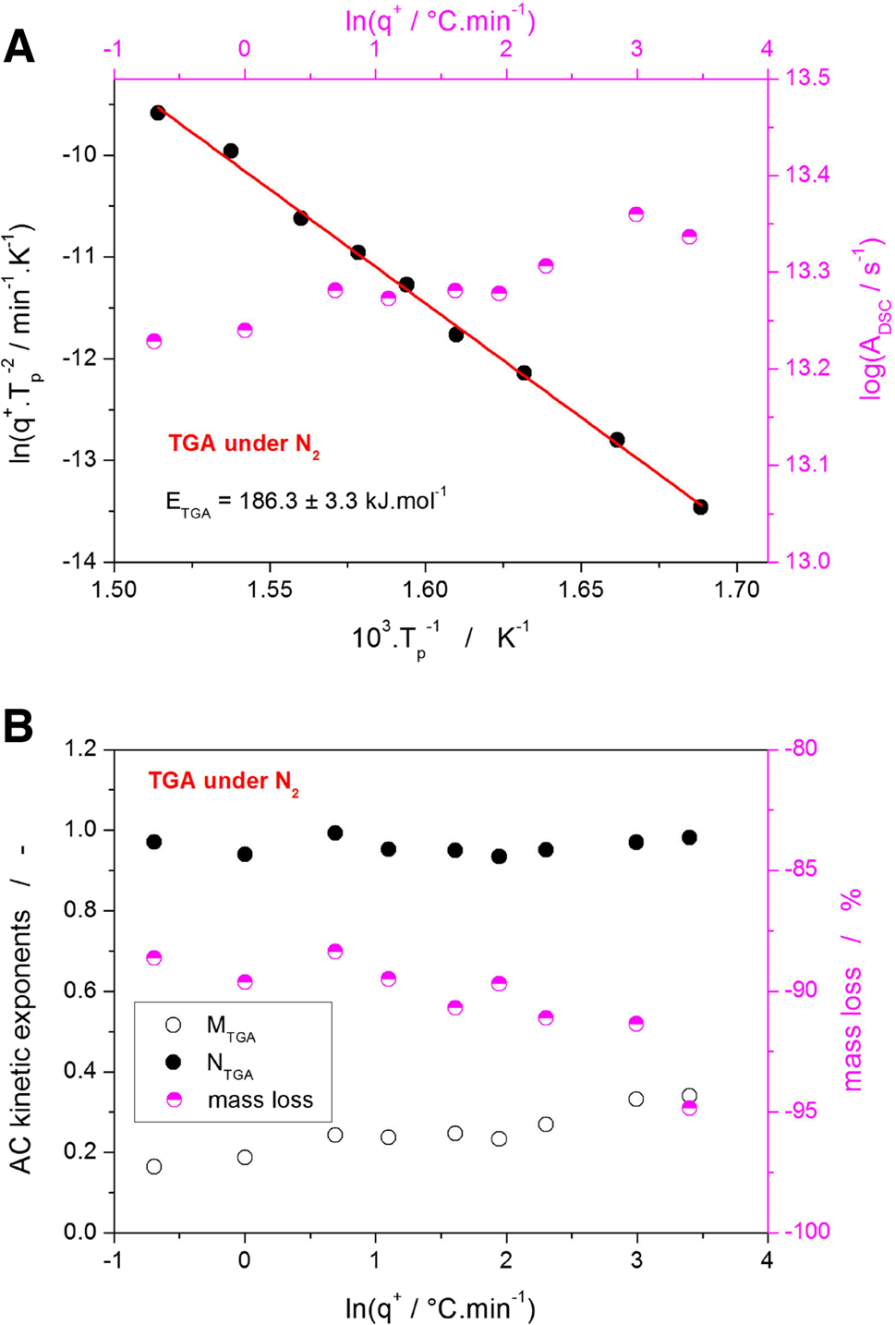
**A** Kissinger dependence for the single decomposition stage measured by TGA in N_2_ – see Fig. 1A. The top and right axes depict the q^+^ dependence of the pre-exponential factor determined by the sc-MKA method. **B** The q^+^ dependences of the M and N kinetic exponents determined by the sc-MKA method for the TGA measurement of the Affinisol powder in N_2_. The right axis shows the scaling for the mass loss data

One also needs to bear in mind that the true reaction scheme is most probably more complicated than introduced in Fig. 8C. Contrary to the degradation in air, under the inert N_2_ atmosphere, the Affinisol decomposes in a single step via first-order reaction with a slight autolytic effect – see Fig. 9. The consistency and uniformity of this primarily solid-state reaction are confirmed by the practically constant values of all kinetic parameters (E, A, M, N, mass loss).

### Kinetic Predictions and Processing Temperatures

The main purpose of the solid-state kinetic analysis is the kinetic predictions of the material behavior extrapolated towards conditions that are difficult or impossible to measure experimentally. The present kinetic predictions will be theoretically simulated for isothermal annealings extrapolated to lower temperatures, where the experiments would be time-demanding, with the experimental conditions being difficult to maintain constant and/or instrumental signals being too weak to record. In accordance with the latest trend in the kinetic predictions [56, 58–60], the extrapolations to low-T annealing can be best done utilizing the set of kinetic parameters obtained for the lowest applied q^+^ (0.5°C·min^−1^ in the present study). Note that the very slow heating is most reminiscent of the isothermal conditions, hence the most accurate kinetic prediction.

**Fig. 10:**
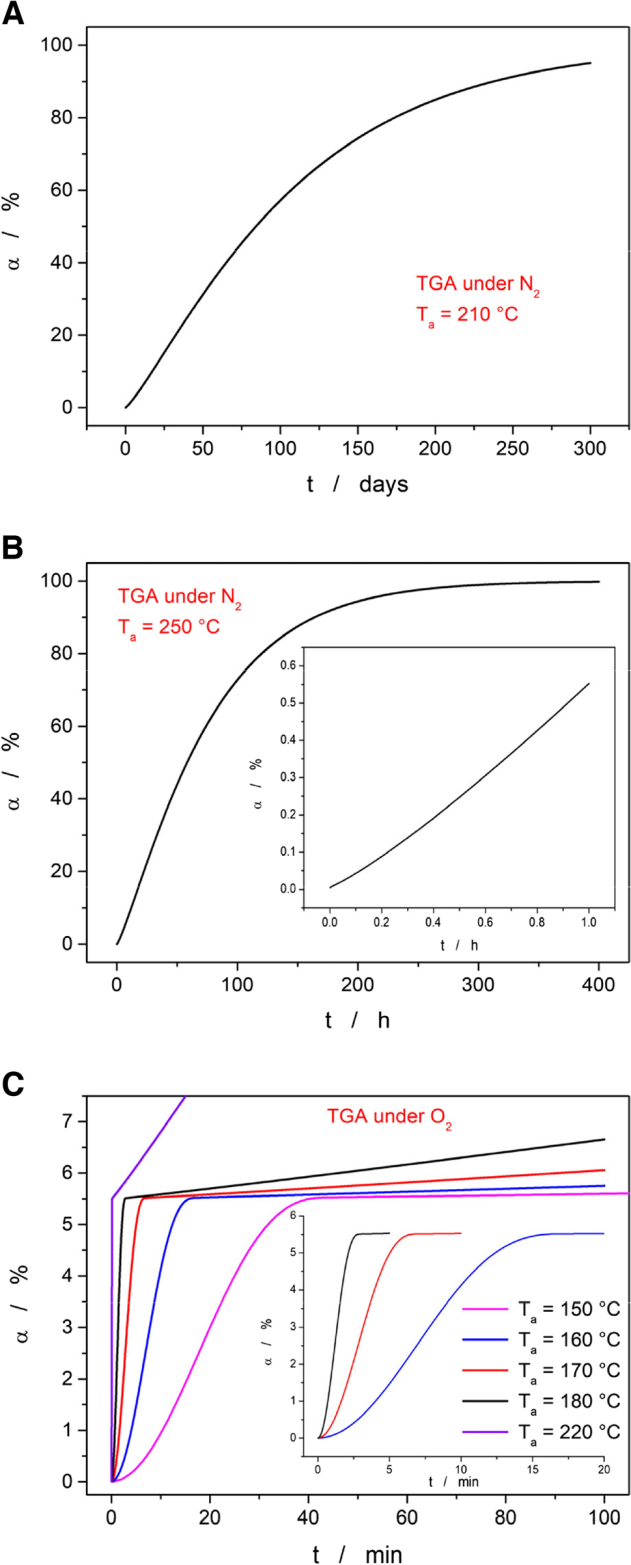
**A** Prediction of the isothermal Affinisol decomposition at 210°C. The calculation was based on the kinetic parameters determined for TGA data measured at 0.5°C·min^−1^ for the Affinisol powder in N_2_. **B** Prediction of the isothermal Affinisol decomposition at 250°C (the inset shows the part of the curve zoomed-in on the first hour). The calculation was based on the kinetic parameters determined for TGA data measured at 0.5°C·min^−1^ for the Affinisol powder in N_2_. **C** Predictions of the isothermal Affinisol decomposition at selected temperatures (the inset shows the measurements at higher T). The calculations were based on the kinetic parameters determined for TGA data measured at 0.5°C·min^−1^ for the Affinisol powder in the air. Note that all the predictions were calculated for the whole reaction scheme (see Fig. 8C), but the graph is zoomed-in on the initial decomposition stage – accounting for the 5.5% mass loss at 0.5°C·min^−1^

Starting with the technologically less important case of the decomposition in the N_2_ atmosphere, the kinetic description obtained for the single decomposition step recorded by TGA at 0.5°C·min^−1^ was used to simulate the rate of Affinisol degradation at temperatures 210 and 250°C (with the former being the highest recommended by the DuPont manufacturer). As is apparent from Fig. 10A, the degradation in the inert N_2_ atmosphere indeed proceeds extremely slowly at 210°C (with the conversion of the first 10% taking ~20 days). At 250°C (see Fig. 10B), the decomposition reaction is significantly accelerated, but it is still well within all possible safe limits applicable for the Affinisol processing (0.5% mass loss after ~1 h). However, a completely different situation arises for the Affinisol degradation in the air – see Fig. 10C.

The first decomposition stage (accounting for approx. 5.5% of the overall mass) is at higher temperatures of T ≥ 170°C completed within a few minutes, i.e., within the timeframe of the estimated Affinisol retain time in the extruder. Note that this finding is in good correspondence with the micrographs from Fig. 5, where the first significant changes are observed for the filament extruded at 170°C. The main dataset in Fig. 10 also indicates that at 220°C, the main decomposition process accounting for approx. 70% of the overall mass loss already proceeds to a significant degree (> 1%) within the first five minutes, which may correspond to the abrupt darkening of the Affnisol filament.

**Fig. 11:**
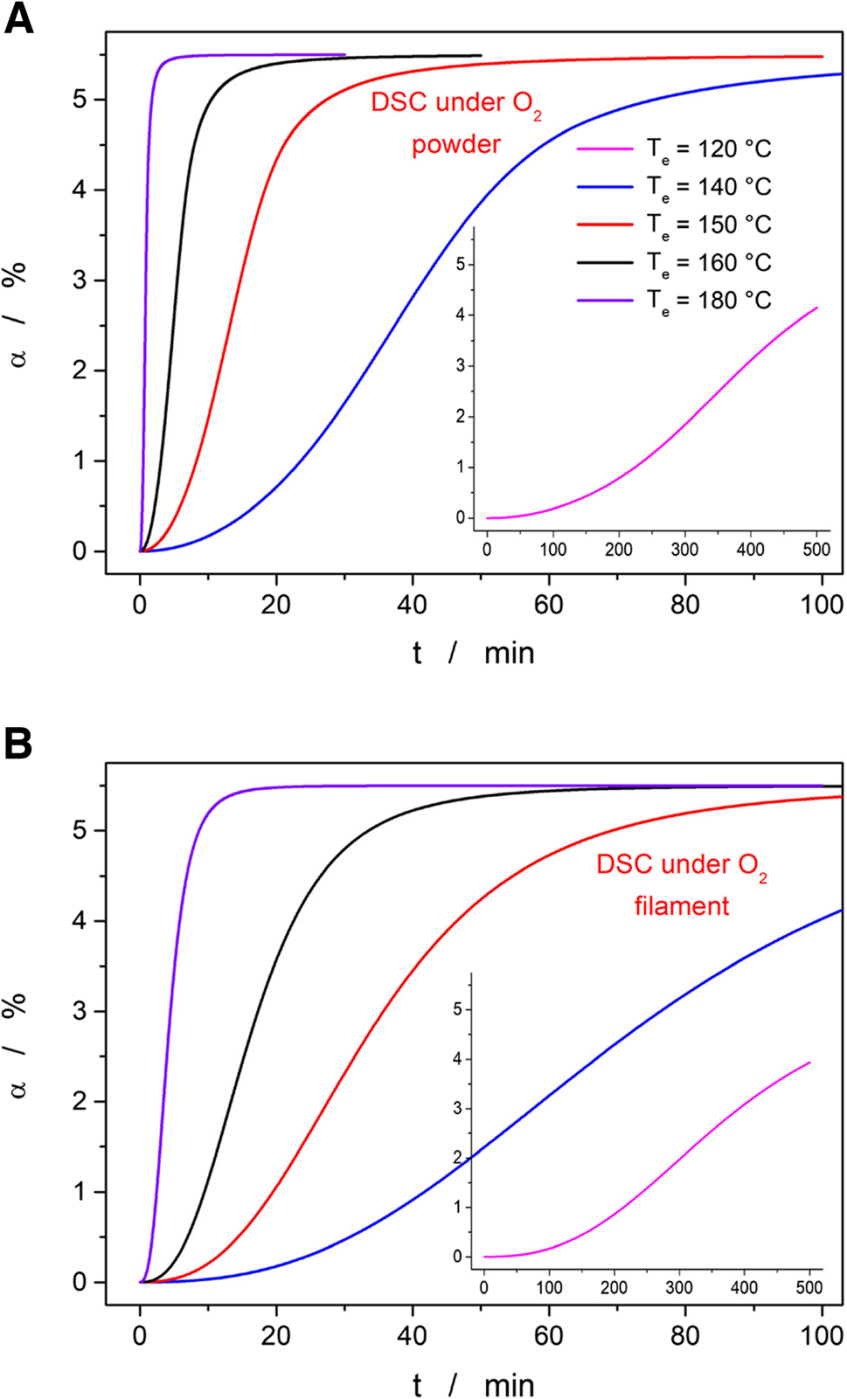
**A** Predictions of the isothermal Affinisol decomposition at selected temperatures (the inset shows the measurement at lowest T). The calculations were based on the kinetic parameters determined for DSC data measured at 0.5°C·min^−1^ for the Affinisol powder in the air. **B** Predictions of the isothermal Affinisol decomposition at selected temperatures (the inset shows the measurement at the lowest T). The calculations were based on the kinetic parameters determined for DSC data measured in air at 0.5°C·min^−1^ for the Affinisol filament extruded at 120°C

Regarding the predictions from the DSC data, the degree of conversion α calculated for the first stage (the only stage that was accurately measured calorimetrically) was arbitrarily set to the same value of 5.5% as obtained from the TGA data. This was done to avoid potential confusion of the readers and also for the lack of other options (since the experimental data to determine the overall enthalpy change are missing). Based on the DTA data measured during the joint STA (TGA + DSC; see Fig. 3C) experiments, the true share of the evolved heat is close to the 7% value, at least at higher q^+^. The predictions based on the kinetic description of the DSC curves obtained for the Affinisol powder and filaments are shown in Fig. 11A and B, respectively. As can be expected from a visual comparison of the raw data (see Figs. 2C and 4B), the degradation/oxidation process in the extruded filaments proceeds significantly slower. This is indeed reflected also in the kinetic predictions – note the intentionally similar scaling of Fig. 11A and B. The predictions based on the powdered Affinisol data suggest that the degradation should be within the 5 min timeframe fully completed even at 160°C (and proceeded to a significant degree at 150°C). This predicted behavior is too fast in comparison with the reality observed for the extruded material (see Fig. 5). On the other hand, the predictions calculated based on the DSC data corresponding to the Affinisol filament correctly predict the full completion of the first degradation stage in the 5 min timeframe for T_e_ = 180°C, and a partial one (but still at significant α) for T_e_ = 170°C. Hence, the high-T oxidation/burn during extrusion is clearly diffusion-limited, and significantly more accurate kinetic predictions of this reaction can be obtained from the measurements of the HME-prepared filaments (rather than from measuring the raw, usually powdered or granulated as-purchased material). This finding is crucial for future kinetic studies of the thermally induced oxidation/degradation in extruded materials.

## Conclusions

Thermal degradation of the Affinisol HPMC HME material was studied by means of TGA and DSC. The novel sc-MKA method paired with the flexible autocatalytic kinetic model was used to provide a full kinetic description of the observed degradation processes. Under the inert N_2_ atmosphere, the degradation proceeded in accordance with the literature data and with the specifications declared by the manufacturer: decomposition onset at ~260°C at 0.5°C·min^−1^, single step mass loss of 90–95%, activation energy of the decomposition process E ≈ 185 kJ·mol^−1^. However, these conditions are rarely met in real-life laboratory or industrial practice, where the high-T processing of Affinisol (e.g., hot melt extrusion or 3D printing) usually proceeds in the air. The thermal degradation induced by the presence of O_2_ drastically changes from that in the inert N_2_ atmosphere and shifts the limits for safe utilization/processing of the Affinisol polymer.

In particular, the thermogravimetric mass loss occurs in three stages – preliminary ~5% mass loss at 150°C, main loss of circa 70% mass slowly onsetting at ~200°C, and the final mass loss step of approx. 15% with the onset at 380°C. All these effects were associated with strong oxidation-caused exothermic heat releases. In addition, a second reaction path emerges at higher q^+^ (≥ 3°C·min^−1^), most probably due to the limited rate of O_2_ diffusion into the partially burned Affinisol material; in this secondary competing chain of reactions, the activation energies appear to be very close to those determined for at low q^+^ but the proportion between the second and third (main and final) decomposition steps changes by ~7% in favor of the main decomposition step. Additional investigation by means of DSC has shown that the first preliminary mass loss step (which is technologically most important because it determines the upper-temperature limit for the Affinisol processing) is probably composed of two following reactions: partial split of the hydroxypropyl and methyl groups, and the consequent oxidation of the released CO (exact nature of these two processes still needs further clarification). The activation energy of the rate-determining process is E ≈ 150 kJ·mol^−1^.

Based on the kinetic predictions calculated for the estimated extrusion hot-zone retain the time of 5 min, the significant degree of Affinisol degradation (first/preliminary decomposition stage reached to α > 20%) was achieved for the extrusion temperatures T ≥ 170°C. This finding was supported also by the visual examination of the color and homogeneity of the HME-processed filaments Affinisol. A very important finding was obtained also regarding the precision of the kinetic predictions: acceptable accuracy was achieved only for the kinetic data of the filament pre-extruded at low T; the powdered Affinisol decomposed significantly faster, and the corresponding predictions should be taken rather as a worst-case scenario than as a true approximation of the first stage of the decomposition process during HME or 3D printing.

To conclude, the proper temperature limit for the laboratory/industrial thermal processing of the Affinisol HPMC HME 15LV polymer is 160°C (up to 5 min exposition under the air atmosphere). This temperature should be sufficient for the hot melt extrusion, where acceptable compactness and textural and mechanical properties can be obtained even at T_e_ = 120°C. For HME processing of Affinisol in pharmacy, the attention thus technically needs to be paid only to the requirements of the added excipients or the incorporated drug. On the other hand, the 3D printing process from the Affinisol filament requires temperatures close to 200°C [23], where not only the initial/preliminary decomposition step is fully completed, but also the main decomposition can proceed to a non-negligible degree. Hence, further research needs to be aimed at the identification of the potential harmful/carcinogenic products of the Affinisol decomposition that can get trapped within the HME-processed or 3D-printed objects. Moreover, the utilization of plasticizers or lubricants decreasing the viscosity of the Affinisol-based formulation (ideally below 170°C) should be considered and explored for 3D printing applications.

## Data Availability

The raw/processed data required to reproduce these findings cannot be shared at this time as the data also forms part of an ongoing study. Selected data may be provided on request.
